# Estimating the impact post randomization changes in staff behavior in infection prevention trials: a mathematical modeling approach

**DOI:** 10.1186/s12879-017-2632-1

**Published:** 2017-08-03

**Authors:** Eric T. Lofgren

**Affiliations:** 1 Paul G. Allen School for Global Animal Health, Washington State University, 240 SE Ott Road, Room 311, Pullman, WA 99164-7090 USA; 2 Community Health Analytics Initiative, Washington State University, Pullman, WA USA

**Keywords:** MRSA, Hospital epidemiology, Healthcare-associated infections, Contact precautions, Mathematical modeling

## Abstract

**Background:**

Randomized controlled trials (RCTs) of behavior-based interventions are particularly vulnerable to post-randomization changes between study arms. We assess the impact of such a change in a large, multicenter study of universal contact precautions to prevent infection transmission in intensive care units.

**Methods:**

We construct a stochastic mathematical model of methicillin-resistant *Staphylococcus aureus* (MRSA) acquisition in a simulated 18-bed intensive care unit (ICU). Using parameters from a recent study of contact precautions that reported a post-randomization change in contact rates, with fewer visits observed in the treatment arm, we explore the impact of several possible interpretations of this change on MRSA acquisition rates.

**Results:**

Scenarios where contact precautions resulted in less patient visitation resulted in a mean decrease in MRSA acquisition rate of 37%, accounting for much of the effect reported in the trial.

**Conclusions:**

Behavior changes that impact the contact rate have the potential to drastically alter the results of RCTs in infection control settings. Careful monitoring for these changes, and an assessment of which changes will likely have the greatest impact on the study before the study begins are both recommended.

**Electronic supplementary material:**

The online version of this article (doi:10.1186/s12879-017-2632-1) contains supplementary material, which is available to authorized users.

## Background

The randomized controlled trial is often considered the “gold standard” study design for interventional studies. The process of randomization removes any differences between the treatment and control arms of the trial, yielding a theoretically unbiased estimate of a causal effect. But this protection only applies to differences between the treatment and control arms that arise *before* randomization – differences that arise afterward, such as differential rates of dropout between the arms can bias this effect.

In multi-center trials of hospital-level policy interventions these post-randomization changes can arise not only from differences in the patients between the two arms, but differences in the behavior of healthcare personnel at different sites [1]. If these differences in behavior result in differential quality of care between the two study arms, bias can arise. This problem is especially pernicious in infectious disease epidemiology [2], as each patient who is infected (or whose infection is prevented) alters not only their own outcome but the outcome of other patients in the same facility, amplifying the impact of what may be fairly subtle differences. A failure to detect and quantify these effects can potentially lead to errant clinical practice, hospital policy, and professional guidelines.

Mathematical models are ideal for assessing whether these post-randomization changes are largely harmless, or serious threats to the validity of a study [3]. By simulating the study population in a counterfactual scenario where only the behavior change (and not the intervention) occurred, they can quantify how much of the joint effect of both the intervention and the behavior change is due to spurious factors, rather than the true effect.

Here, as a motivating example, we examine the impact of a post-randomization change in the frequency at which healthcare workers (HCWs) reported visiting patients on a major multicenter clinical trial of universal glove and gown use on the acquisition of antibiotic resistant organisms [4]. This study, which is the largest of its kind examining the use of gowning and gloving, took place between January and October 2012 and involved 20 medical and surgical intensive care units (ICUs). Healthcare workers were required to wear gloves and gowns for all patient contact and when entering a patient’s room, rather than following these precautions only for patients known to have infection or colonization with antibiotic resistant bacteria, per CDC guidelines [5]. This study found that the use of a universal gowning and gloving policy reduced MRSA acquisitions by 40.2%, reflecting 2.98 fewer acquisitions per 1000 patient days (95% Confidence Interval (CI): 5.58 to 0.38).

In Harris et al., the authors report 0.96 fewer healthcare worker visits per hour in the intervention arm of the trial, as well as a rise in hand-hygiene compliance on room exit in the intervention arm. Both of these behavior changes may alter the estimated effect of the intervention, potentially dramatically reducing the reported effectiveness of universal gowning and gloving. To date, this impact has not been quantified, and the magnitude of the potential bias (and thus the effectiveness of the intervention) remains largely speculative and rooted in personal opinion and anecdote. A mathematical model of methicillin-resistant *Staphylococcus aureus* (MRSA) acquisition in an ICU was used to examine the magnitude of the potential bias present in the study, modeling the change in visitation and hand-hygiene with no concurrent gowning or gloving based intervention.

## Methods

### Transmission model

**Fig. 1:**
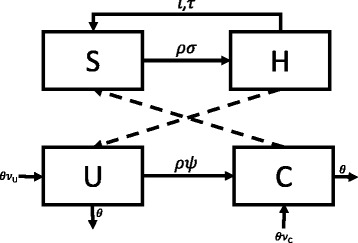
Schematic representation of the compartmental flow of a mathematical model of methicillin-resistant *Staphylococcus aureus* (MRSA) acquisition in an intensive care unit. *Solid arrows* indicate possible transition states, while *dashed lines* indicate the potential routes of MRSA colonization/contamination. Healthcare workers are classified as uncontaminated (S) or contaminated (H), while patients are classified as uncolonized (U) or colonized (C). Greek characters represent the parameters governing each transition

The transmission of MRSA through an ICU was modeled as a series of compartments representing patient health, as well as whether or not a healthcare worker’s hands were presently contaminated with MRSA (Fig. 1). HCWs were modeled as being either uncontaminated (S) or presently carrying MRSA on their hands or gloves (H). Patients were divided into two states – those not presently colonized with MRSA (U) and those colonized (C). The interactions between these compartments was governed by a series of eight stochastic transitions, the details of which are presented in Table 1. The recorded outcome was incident MRSA colonization (i.e. transitions from U to C).

**Table 1:** Transitions and Parameters for a Mathematical Model of MRSA Acquisition in an Intensive Care Unit

Transition	Equation	Parameter description	Parameter value	Source
H to S	*ιH*	ι: Effective hand-decontaminations per hour (# of direct care tasks × hand hygiene compliance × efficacy)	ι: 5.74 (10.862 direct care tasks × 56.55% compliance × ~ 95% efficacy)	ι: [4, 8, 9, 20]
H to S	$\tau H\frac{C}{C+U}$	τ: Effective gown/glove changes per hour (2 × # of visits × compliance)	τ: 2.389 (2.89 changes per hour × 82.66% compliance)	τ: [4, 21]
S to H	$\rho \sigma C\frac{S}{\left(S+H+C+U\right)}$	ρ: Contact rate between patients and HCWs. σ: Probability that a HCW’s hands are contaminated by contact with a colonized patient or their environment	ρ: 4.154 direct care tasks/h σ: 0.054	ρ: [8, 9] σ: [21]
U to C	$\rho \psi U\frac{H}{\left(S+H+C+U\right)}$	ψ: Probability of successful colonization of an uncolonized patient due to contact with a contaminated HCW.	ψ: 0.0931	ψ: Fitted to [4]
U Discharge to U Admission	*θν*_*U*_ *U*	θ: Probability of discharge (1/average length of stay) ν_U_: Proportion of admissions uncolonized with MRSA	θ: 0.00949 ν_U_: 0.922	θ: [4] ν_U_: [4]
U Discharge to C Admission	*θν*_*C*_ *U*	ν_C_: Proportion of admissions colonized with MRSA	ν_C_: 0.078	ν_C_: [4]
C Discharge to U Admission	*θν*_*U*_ *C*			
C Discharge to C Admission	*θν*_*C*_ *C*			

Because there is evidence that MRSA can be transmitted by surface-contamination as well as direct contact [6], surface contamination is indirectly modeled by defining contact between patients and HCWs as direct care tasks [7, 8], which could involve either interaction with the patient, or interaction with the environment near the patient. The model makes several simplifying assumptions. First, all HCWs are assumed to visit all patients – there is no cohorting or other individual assignment of HCWs to particular patients. Patients were assumed not to interact with each other and were assumed to be in single-occupant rooms. Hospitals were assumed to follow standard contact precaution guidelines set forward by the CDC and to detect MRSA colonization with perfect accuracy, and colonization was assumed to be permanent. Finally, all HCWs are assumed wash or decontaminate their hands after each direct care task, and change their gloves and/or gowns on entry and exit to a patient room. These assumptions are intended to examine the effect of the contact rate changes in settings that are otherwise largely free of major failings in their infection control programs.

### Parameterization and model calibration

As the raw data from Harris et al. is not publically available, the model described in the previous section was parameterized using data drawn from the literature. The values of each parameter, and the source they were drawn from, are described in Table 1, with a differential equation representation of the model available in Additional file 1. In many cases, parameters were drawn from the study itself or from studies conducted at one of the hospitals participating in the trial [9, 10]. These parameters are designed to mimic the control-arm of the trial, save for the change in hand hygiene and patient contact rates. This allows for the isolation of the impact of these changes separate from those of direct effect of the intervention.

A single parameter (ψ), the probability that contact between a contaminated healthcare worker and a uncolonized patient would result in effective colonization, was used to calibrate the model. Approximate Bayesian Computation (ABC) [11] was used to obtain a Bayesian posterior estimate of this parameter that matched the MRSA incidence rate in the model with that in the original study. Specifically, a candidate parameter value was drawn from a uniform distribution bounded by 0 and 0.20, and the model system was simulated with this parameter 25 times. The candidate parameter was accepted if the average incidence of these 25 model runs was within the 95% confidence interval of the original study’s MRSA acquisition rate in control hospitals during the study period (4.59 to 7.67 cases/1000 patient days) [4]. This process was then repeated 1000 times to yield a distribution of accepted candidate parameters, which also an approximation of the Bayesian posterior for the parameter. The median of this distribution was then used as the value for ψ, yielding a modeled incidence density similar to that of the parent study.

### Modeled scenarios

The model was used to simulate the transmission of MRSA in a single 18-bed ICU (the average ICU in the trial) staffed by six nurses and a single dedicated intensivist. Each time a patient was discharged, another patient immediately took their place to maintain a fixed ICU population [12]. Five scenarios were considered – first, a baseline scenario where no behavior change took place and the contact rate between patients and HCWs remained constant. Second, a scenario where the number of visits decreased and proportionally fewer direct care tasks were performed, representing HCWs actively avoiding visiting patients due to the inconvenience of having to don gowns and gloves as part of the intervention. This is modeled as a 17.7% reduction in overall contact, the weighted average of the reduction in contact between those in the treatment arm and those in the control arm (both on and off contact precautions). Third, a scenario where the number of visits went down by 17.7%, but the number of direct care tasks remained the same, representing HCWs trying to pack more tasks into a single visit. This scenario is modeled as more tasks being performed before the HCW has an opportunity to change out of their gown and gloves, increasing the risk of contamination. Finally, the last two scenarios considered the 2nd and 3rd scenario in the presence of an increase in the hand-hygiene compliance rate to 64.25% (the weighted average of entry and exit hand-hygiene compliance reported in the trial’s intervention arm).

Each scenario was stochastically simulated 1000 times in order to capture the variability in the system using Gillespie’s Direct Method [13]. This gives the model two important properties. First, individuals within the model are treated as discrete units, which prevents small fractions of individuals existing in any compartment. Second, because individuals are treated this way and the model is probabilistic, we can capture variability in the system due to random chance, which is important for understanding the underlying dynamics of infectious diseases in small populations. The models were run for a full year with each time step in the model representing a single hour.

### Statistical analysis and parameter sensitivity

The primary outcome of interest was the rate of hospital-attributable MRSA colonizations (i.e. those whose infection source was within the hospital) occurring over the course of the simulation. In order to assess the sensitivity of the model’s findings to errors in parameter values or model structure, two types of sensitivity analysis were performed. First, the baseline scenario and both types of behavior change (absent a co-occurring change in hand hygiene compliance) were re-run, allowing all parameters to vary uniformly within ±20% of their original value. Second, the impact of a change in contact rates was explored over a much wider range of values (0% to 50%) for both types of behavior changes (absent a co-occurring change in hand hygiene compliance) by simulating both scenarios 5000 times randomly drawing the contact rate change from a uniform distribution ranging from a 0% reduction to a 50% reduction, rather than being fixed at 17.7%, creating a data set of contact rates and their corresponding incidence rates. The results of this analysis were analyzed using a Poisson regression model to provide an incidence density ratio (IDR) reflecting the expected increase or decrease in the MRSA acquisition rate corresponding to a 1% chance in contact rate.

All simulations were performed in Python 2.7 using the StochPy library [14], and all statistical analysis was performed in R version 2.15. The code and simulation results are available online at https://github.com/epimodels/contactchange.

## Results

### Model calibration and baseline

**Fig. 2:**
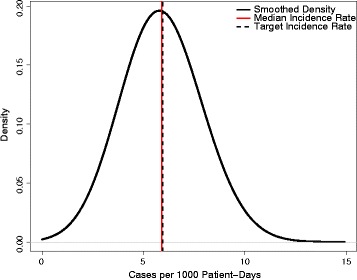
Calibration results of a mathematical model of methicillin-resistant *Staphylococcus aureus* (MRSA) acquisition in an intensive care unit. The *solid black line* represents the kernel-smoothed incidence density of 1000 runs of a stochastic simulation, with the *solid vertical red line* showing the median of this distribution and the *dashed black line* the rate reported in the original trial

**Fig. 3:**
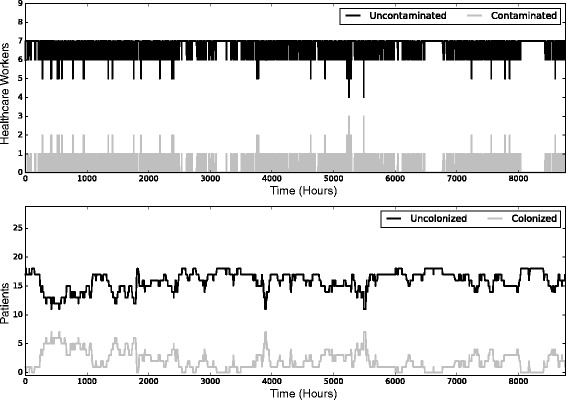
A single stochastic realization of a mathematical model of methicillin-resistant *Staphylococcus aureus* (MRSA) acquisition in an intensive care unit. The *top panel* shows the level of hand contamination in healthcare workers, while the *bottom panel* depicts the number of colonized and uncolonized patients, both over a 1-year period

The fitted model produced an average incidence density of 5.89 acquisitions per 1000 patient-days (standard deviation (SD) = 1.35), slightly lower than the trial’s reported rate of 5.94 cases per 1000 patient-days, but well within the bounds of expected variability (Fig. 2). Overall, the model produced results in line with the “typical” transmission of MRSA in hospitals – low levels of MRSA colonization, many of which involve patients who were colonized when admitted, with the occasional more serious, hospital-driven outbreak. Contamination of healthcare worker hands was present at low levels, but this contamination was commonly transient, with no long-term sustained hand contamination. A time-series of a single exemplar run of the model is shown in Fig. 3.

### Behavior change scenarios

**Fig. 4:**
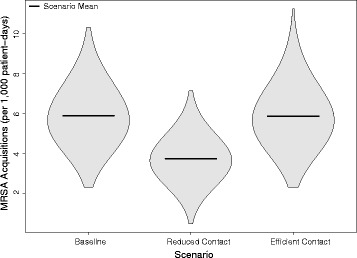
Violin plot of the outcome of 3000 simulations of methicillin-resistant *Staphylococcus aureus* (MRSA) acquisition in an intensive care unit, showing the mean and probability density of MRSA acquisitions per 1000 patient-days in each of the three main simulated scenarios

Compared to the baseline, no-intervention scenario, the decrease in visits resulting in less contact resulted in a 36.9% decrease without an accompanying hand hygiene compliance, or a mean of 3.72 acquisitions per 1000 patient-days (SD = 0.97). If instead the amount of patient contact remained the same, but patient care tasks were grouped into fewer visits, the acquisition rates remained essentially unchanged with a 0.2% decrease or an average of 5.87 acquisitions per 1000 patient-days (SD = 1.39). The results of the three scenarios without a change in hand hygiene is shown in Fig. 4. The observed change in hand-hygiene compliance co-occurring with each scenario increased the resulting reductions to 3.18 acquisitions per 1000 patient-days (SD = 0.86) and 5.08 acquisitions per 1000 patient-days (SD = 1.23) respectively.

### Sensitivity analysis

**Fig. 5:**
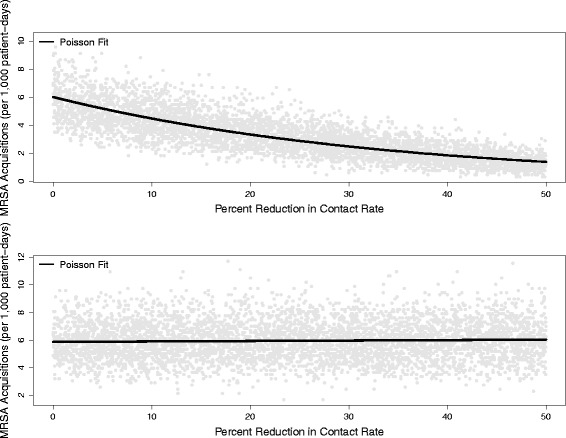
The estimated impact of a reduction on contact rates between healthcare workers and patients on the acquisition of methicillin-resistant *Staphylococcus aureus* (MRSA). The *top panel* depicts the rate of MRSA acquisition per 1000 patient-days emerging of 5000 simulations of randomly drawn reductions in contact rate ranging from 0% to 50%, assuming this reduction directly results in less contact with the patient. The *lower panel* depicts the same outcome, assuming this reduction results in the same amount of care, but with more tasks being compressed into a single visit. *Light grey circles* are the result of a single simulation run, while the *black line* is the predicted fit of a Poisson regression model for both scenarios

The sensitivity analysis showed broadly similar findings (double-digit percentage change for the reduced contact scenario and a negligible difference for the efficient contact scenario) as the reported results when allowing for parameter uncertainty. For the wider parameter sweep, a one-unit decrease in contact rate for the reduced contact scenario corresponded to an incidence rate ratio (IRR) of 0.97 (95% CI: 0.97,0.97), while for the efficient contact scenario it resulted in an IRR of 1.00 (95% CI: 1.00,1.00). While both were statistically significant at *p* > 0.001, the latter is of questionable clinical impact. These simulation results and the corresponding regression fit are shown in Fig. 5.

## Conclusions

If the reduction in HCW visits reported in Harris et al. represents a reduction in patient contact, then the observed reduction in MRSA acquisitions can be almost entirely explained by the post-randomization changes in HCW visitation rates and hand hygiene. Universal gowning and gloving may in fact have much a much more modest impact on MRSA acquisitions, but the true effect cannot be known without further investigation of the behavior change itself. Hospitals considering the implementation of universal gowning and gloving protocols should consider whether a smaller anticipated effect still meets their infection control objectives.

Whether the post-randomization behavior changes are themselves desirable remains an open question. While improved hand hygiene rates are beneficial for the prevention of healthcare-associated infections, the desirably of decreased visitation rates is less obvious. There are concerns that fewer visits by healthcare workers might result in poorer care and a lower likelihood of adverse events being thwarted by vigilant clinical staff [15, 16], though Harris et al. found no increase in adverse events between the treatment and control arm, and this concern remains controversial [17]. It is also possible that fewer visits – without a corresponding decrease in care – might be beneficial to patients, reducing unnecessary disruptions to their schedules and sleep [18]. One might argue that the standard intent-to-treat (ITT) analysis used in Harris et al. incorporates this post-randomization change, however if the majority of the effect seen in the ITT analysis is, as this model suggests, the result of unintended effects of the intervention, it is possible that similar gains may be made without the need for an expensive and time-consuming universal gowning and gloving intervention.

This study is not without limitations. As with all mathematical models, the model’s structure and assumptions heavily influence its results. Particularly, this model’s parameters are drawn from a number of different data sources, rather than being a direct computational simulation of the original study from its own data. It is likely however, even with the original data, that a large number of parameters would need to be derived from the literature. Where possible, parameters have been drawn from studies similar to the setting of the original study to minimize the divergence between the study’s hospitals and those simulated in the model. Furthermore, allowing all the parameters in the model to vary produced similar findings, suggesting the model is robust to a reasonable degree of uncertainty. The findings of the model remain consistent over a broad range of changes in contact rate and should be applicable beyond the single motivating example of Harris et al. The results of the model are not an attempt to estimate a precise effect or adjustment to the original study by Harris et al. Rather, it uses this study as a motivating example (partially due to the richness of results presented), to demonstrate the magnitude of confounding that a post-randomization change in contact rates poses to a study of this type.

More broadly, this study illustrates the growing need for the involvement of experts in infectious disease modeling at the outset of a study’s design, similar to the role biostatisticians play today. As interventions grow more sophisticated and involve complex behavioral changes, the use of dynamic models can identify critical aspects of disease transmission that may affect the outcome of the study. These can then be accounted for, or at the very least measured, in order to disentangle the study’s primary effect from co-occurring behavioral changes. These models may also be able to identify where a proposed intervention is unlikely to succeed at all [9, 19]. By combining infectious disease dynamics with conventional observational studies and clinical trials, we can strengthen the overall evidence produced by these studies, critically evaluate alternative explanations for their observed effects, and avoid foreseeable errors in the design and evaluation of interventional studies.

## Additional file

Additional file 1: Ordinary differential equation representation of a mathematical model of MRSA acquisition in an intensive care unit. (DOCX 76 kb)

## Abbreviations

ABC: Approximate Bayesian Computation
CDC: Centers for Disease Control and Prevention
CI: Confidence Interval
HCW: Healthcare worker
ICU: Intensive care unit
IDR: Incidence Density Ratio
ITT: Intent-to-Treat
MRSA: Methicillin-resistant *Staphylococcus aureus*
RCT: Randomized controlled trial
SD: Standard deviation
