# Supplementary material for: Estimating the impact post randomization changes in staff behavior in infection prevention trials: a mathematical modeling approach
Source: BMC Infect Dis. 2017 Aug 3;17:539. doi: 10.1186/s12879-017-2632-1 (PMC5541411; doi:10.1186/s12879-017-2632-1)
Supplement: Additional file 1: — Ordinary differential equation representation of a mathematical model of MRSA acquisition in an intensive care unit. (DOCX 76 kb) [file 12879_2017_2632_MOESM1_ESM.docx]

Estimating the Impact Post Randomization Changes in Staff Behavior in Infection Prevention Trials: A Mathematical Modeling Approach

**Ordinary differential equation representation of a mathematical model of MRSA acquisition in an intensive care unit**

The transitions and parameters used in the stochastic simulation model discussed in the main manuscript may be represented as a series of ordinary differential equations to provide a representation of how these transitions interact, and to allow an easier comparison with other ODE-based models in the literature.

$$\frac{dS}{dt}=\iota H+\tau H\frac{C}{C+U}-\rho\sigma C\frac{S}{(S+H+C+U)}$$

$$\frac{dH}{dt}=\rho\sigma C\frac{S}{(S+H+C+U)}-\iota H-\tau H\frac{C}{C+U}$$

$$\frac{dU}{dt}= \theta\nu_{U}U+\theta\nu_{U}C- \theta\nu_{C}U-\theta\nu_{U}U-\rho\psi U\frac{H}{(S+H+C+U)}$$

$$\frac{dC}{dt}= \rho\psi U\frac{H}{(S+H+C+U)}+\theta\nu_{C}U+\theta\nu_{C}C-\theta\nu_{C}C-\theta\nu_{U}C$$

Note that due to the circular nature of some discharge/admission events that keep the system at a steady-state, discharges and admissions into the same compartment (i.e. a U discharge leading to a U admission) are represented twice in each equation. These would naturally cancel out, however they have been retained in the equation representation for clarity.
